# The Impact of Personality in the Selection of Teacher Students: Is There More to it Than the Big Five?

**DOI:** 10.5964/ejop.v14i3.1536

**Published:** 2018-08-31

**Authors:** Corinna Koschmieder, Barbara Weissenbacher, Jürgen Pretsch, Aljoscha C. Neubauer

**Affiliations:** a Department of Differential Psychology, University of Graz, Graz, Austria; Department of Psychology, Webster University Geneva, Geneva, Switzerland; Università di Roma LUMSA, Rome, Italy

**Keywords:** personality, bandwidth-fidelity dilemma, longitudinal study, academic success, teacher students

## Abstract

The bandwidth-fidelity dilemma is a controversially discussed problem in personality measurement. In this study, we contrasted the utility of broad versus narrow personality traits in an admission exam for teacher students. We compared the Big Five and narrow personality constructs (social-communicative behavior, achievement behavior, health and recreation behavior), which were part of an assessment battery for teacher student selection (N = 1120), regarding overlap and predictive validity. As criterion variables, academic satisfaction (N = 184) and GPA (N = 680) were assessed later. Reasonableness of including both questionnaires in one assessment may be questioned in terms of overlap of the personality inventories. Results show that health and recreation behavior cannot be covered by the Big Five in a selection procedure. Empirically, both broad and narrow traits show predictive validity for academic success and satisfaction.

The bandwidth-fidelity dilemma states that the bandwidth of a predictor has influence on the criterion validity. In most assessments, not only broad criteria, such as overall work success, are of interest but also narrow criteria, such as burnout. This is the case in a newly developed teacher student assessment in which two bandwidth-wise different personality measures were used. Keeping economical testing in mind, are both measures needed?

## Teacher Student Selection

If access to a certain academic discipline has to be restricted, suitable selection criteria and processes need to be established. But what could these criteria be? First, it is obvious that only people who are likely to succeed at university should be admitted. Therefore, traits that are essential for academic success should be considered. But how do we define *success*? On the one hand, people who perform well (e.g. achieve a high GPA) may be regarded as successful. On the other hand, success can also be interpreted in terms of study satisfaction (Rindermann & Oubaid, 1999). Second, the expected job success must be taken into account. When someone enrolls in teacher education, we can assume that this person aims to become a teacher (Mayr, 2012). In other words, when selecting candidates applying for teacher education, it seems important to not only predict whether they will be successful in their studies but also whether they have the potential to be good teachers. Recently, a new selection tool for teacher students was developed, which has been used since in almost all Austrian institutions offering teacher education (Neubauer et al., 2017). The selection tool involves three steps; here, we only examine the second part, namely a psychometric test battery.

Neubauer et al. (2017) showed that individual performance on this test battery accounted for 18% of variance in academic achievement in the first semester, for 15% of variance in academic satisfaction and for even 28% of variance in the intention to quit after four months in college. Most of the variability was explained by linguistic knowledge/ability and by emotional competencies as well as by certain personality traits. The teaching profession can be described as one of the most exhausting ones involving high levels of emotional stress. Therefore, personality traits are of particular importance for teachers’ health and associated job satisfaction. For this reason, selection processes for future teachers should aim at finding students whose personality fits the teacher profession and who are properly equipped to manage the challenges of this profession (Schaarschmidt, 2005). Although the use of self-report personality measures for selection purposes has been discussed controversially (e.g. Klassen & Tze, 2014), the current admission exam indicates that the validity of personality testing is not substantially limited by faking (Krammer, Sommer, & Arendasy, 2017).

## The Impact of Personality on Teachers’ Academic and Job Success

If we address the impact of personality traits on teaching success, previous research has not shown consistent results. Numerous researchers found only weak relationships between personality traits and teaching performance and therefore conclude that “teachers are made, not born” (Klassen & Tze, 2014, p. 73). However, other researchers demonstrated the importance of personality in the context of academic success and actual job success in the teaching profession. Hanfstingl and Mayr (2007) meta-analyzed associations between the Big Five factors and different criteria of academic and work success of (future) teachers. They concluded that the impact of personality on the teaching profession rarely differed from other professions: conscientiousness and emotional stability were particularly important. Extraversion emerged as a valid predictor of job performance and satisfaction but not of academic success. Openness and agreeableness showed only weak or moderate correlations with selected aspects of academic and work success.

A comprehensive longitudinal study about the teaching profession in Germany discovered some narrow traits that are counterproductive for job success: poor resistance, poor social-communication skills and poor self-confidence (Schaarschmidt, 2005). Moreover, it was concluded that these traits could not be trained adequately within a few years of education studies and thereforehad to preexist when entering teachers’ college. One of the key traits in preventing burnout and dropout in teachers is *health and recovery behavior* (Rudow, 1994).

## Using Broad and Narrow Traits in a Selection Procedure

The question of whether broad or narrow traits are preferable for a certain assessment purpose has been discussed for a long time under the label *bandwidth-fidelity dilemma* (Cronbach & Gleser, 1965). It examines the question of whether personality assessments should rather focus on lower-level narrow traits or on higher-order broad traits. If narrowly defined personality traits are assessed, the measurement reliability is usually higher than when broadly defined traits are used. However, narrow traits also have a big disadvantage: they only cover a small area of behavior. This means that the fidelity is higher if narrow traits are measured but that the bandwidth is higher if broad traits are measured.

Ones and Viswesvaran (1996) recommend the use of broad traits for personnel selection purposes due to the advantage in predicting *overall job performance*. However, Jenkins and Griffith (2004) suggest identifying relevant narrow traits for a specific job because in their view, they predict performance better than broad traits and should therefore be used in personnel selection.

## The Present Study: Comparing Broad and Narrow Traits

In the aforementioned newly developed test battery (TESAT – Teacher Student Assessment Austria: Neubauer et al., 2017), personality plays an important role. Therein, we assess global (broad) as well as specific (narrow) personality traits; the latter are deemed especially important for situations that future teachers have to cope with. In this study, we focus our attention on the critical question if actually both types of personality traits provide unique contributions. As broad traits, the Big Five factors were assessed (John & Srivastava, 1999). For assessing narrow traits, we used a work-related inventory, the IPS (Inventory for Personality Assessment in Situations; Schaarschmidt & Fischer, 2007). It comprises 15 situational factors, which are assigned to three personality domains. The domain *social and communicative behavior* comprises the six factors (1) activity in familiar communicative situations, (2) assertiveness when communication is required, (3) tendency to confrontation in social conflict situations, (4) efficacy in a leadership role, (5) considerateness in social responsibility situations and (6) sensitivity to social frustration. Compared to the Big Five model, factors (1), (2) and (4) seem close to extraversion, whereas (3) and (5) could be interpreted similarly to agreeableness and (6) captures aspects of emotional stability.

The domain *achievement behavior* also consists of six factors, namely (7) commitment when high level of performance is required, (8) inertia when change of attitude is required, (9) stability when under stress, (10) self-confidence in test situations, (11) career commitment and readiness to take risks in job-related situations and (12) optimism in the face of everyday demands. Factor (7) appears similar to the Big Five factor conscientiousness, factors (9), (10) and (12) could be matched with emotional stability, (8) comprises aspects of both openness and emotional stability and (11) covers aspects of conscientiousness and extraversion.

The domain *health and recreation behavior* includes the factors (13) ability to relax after the working day, (14) active recreation behavior in free time and (15) preventive health behavior in response to warning signals. These three factors cannot be assigned clearly to corresponding Big Five factors; however, (13) comprises aspects of emotional stability, (14) appears similar to aspects of extraversion and (15) covers aspects of conscientiousness.

The aim of our study is to investigate if both questionnaires are useful. How distinct or redundant are the constructs measured? Are both constructs necessary to identify the best future teachers?

## Research Questions

The question of which personality traits have to be considered in teacher student selection should not be started at the point of predictive validity but with a clear theory and knowledge about the measured constructs. Based on this view, the main goal is to investigate the relations and differences between the broad and narrow personality constructs.

First, the associations between the broad and the narrow traits will be examined. We propose the following assumptions:

1. *Social and communicative behavior* is highly positively related to extraversion.
2. *Achievement behavior* is correlated positively with conscientiousness and emotional stability.
3. *Health and recreation behavior* should be less related to the Big Five than the other two domains; however, we expect positive associations with emotional stability and conscientiousness.
4. All domains of the IPS show small or zero correlations with agreeableness and openness.

Second, we want to investigate if it is useful to use both constructs, the IPS traits and the Big Five traits. We contrast how much variance of the Big Five can be explained by the narrow personality traits and vice versa. Paunonen (1998) observed that aggregated broad personality traits could be predicted better by lower trait level facets than vice versa. This is linked to the fact that aggregation leads to a loss of specific variance. Based on these findings, we expect higher overlaps of the Big Five factors predicted by the IPS facets than vice versa.

Third, the impact of broad versus narrow personality traits on two criteria of academic success, namely achievement and satisfaction, should be examined. We expect academic achievement to show the highest association with conscientiousness and academic satisfaction to be associated the highest with emotional stability. Additionally, we expect a relationship between the IPS domain *achievement behavior* and academic achievement as well as between *health and recreation behavior* and academic satisfaction. Besides, we want to explore which IPS facets are related to academic success.

## Method

### Sample

The admission exam was conducted at four universities. The total sample consisted of 1120 candidates (675 women and 445 men; ages range from 17 to 49 years; age: *M =* 20.89 years, *SD* = 4.48). Four people had to be excluded due to technical problems during the admission exam. In order to investigate the predictive validity, we used the results of two follow-up samples. Subsample 1 comprised 680 students (408 female, 272 male; age: *M* = 20.00 years, *SD* = 3.06), who took exams in the first two years. Subsample 2 included 184 people (141 female, 43 male; age: *M* = 20.55 years, *SD* = 3.86), who completed an optional follow-up questionnaire.

### Measures

#### Personality

For measuring broad personality traits, the German translation of the BFI-42 (Big Five Inventory; John & Srivastava, 1999; German version by Lang, Lüdtke, & Asendorpf, 2001) was used. The reliability in the present sample was satisfying for all scales (*N* = 1116; openness: α = .81, conscientiousness: α = .81, extraversion: α = .83, agreeableness: α = .70 and emotional stability: α = .78). Narrow personality traits were assessed by the German Inventory for Personality Assessment in Situations (Schaarschmidt & Fischer, 2007). This questionnaire comprises 80 items on 15 factors, which can be assigned to three behavior domains (see introduction section 1.3). Reliabilities range from α = .71 to α = .91. For every factor, a situation with five 4-point Likert type items was presented (e.g. situation: “*I am sitting in a sociable group with friends and acquaintances…*”; Item: “*In such a situation, I will probably be talkative*.”) ranging from “definitely true” to “not true at all”. The five items were aggregated for the factor score. For computing the three domain scores, weighted factor scores were used.

#### Academic Success

Academic achievement was operationalized as the average of all courses completed in the first two years. The grades were obtained from university records. To facilitate the interpretation, the GPA mean score weighted by credits (Krammer, Sommer, & Arendasy, 2016) was recoded so that low numbers indicated poor achievement. Academic satisfaction consisted of three items on a six-point Likert-scale (e.g. “*I am satisfied with my choice of academic study*.”). The scale had a satisfactory reliability of α = .68.

### Procedure

On three days, participants were tested in groups of maximum 250 people, each for three hours. For assessing academic satisfaction, the students were tested in regular college classes four months after the admission exam^i^ as part of a regular lesson. To generate the GPA for the first four semesters, the local student database was used.

## Results

### Relations Between Broad and Narrow Personality Traits

First, the correlations of the broad and the narrow traits were inspected (Table 1). We found moderate to high correlations between the factors of the BFI-42 and the IPS. The correlations of the three IPS domains with the Big Five were mostly higher than the correlations of the 15 IPS facets with the Big Five.

**Table 1:** Descriptive Statistics and Correlations of the Big Five Factors With the IPS Domains and Facets

Parameter and Construct	*M*	*SD*	Openness	Extraversion	Emotional Stability	Conscientiousness	Agreeableness
*M*	–	–	4.09	4.18	2.05	4.20	4.14
*SD*			0.47	0.48	0.52	0.48	0.45
Social and communicative behavior	1.87	1.41	.34**	.70**	.56**	.49**	.40**
1. Activity	16.95	1.86	.18**	.55**	.22**	.18**	.17**
2. Assertiveness	16.45	2.44	.28**	.68**	.42**	.34**	.26**
3. Confrontation	13.70	3.04	-.22**	-.27**	-.42**	-.46**	-.55**
4. Leadership	16.93	2.03	.20**	.35**	.25**	.23**	-.03
5. Considerateness	18.05	1.71	.21**	.22**	.16**	.28**	.41**
6. Sensitivity	11.99	2.47	-.22**	-.39**	-.53**	-.34**	-.30**
Achievement behavior	1.85	1.73	.36**	.55**	.59**	.53**	.39**
7. Commitment	15.13	2.49	.23**	.35**	.33**	.46**	.31**
8. Inertia	9.58	2.29	-.22**	-.37**	-.43**	-.31**	-.28**
9. Stability	16.83	2.22	.28**	.33**	.42**	.37**	.27**
10. Self-confidence	15.26	2.37	.19**	.34**	.49**	.27**	.20**
11. Career commitment	16.97	2.22	.27**	.41**	.33**	.33**	.23**
12. Optimism	17.64	1.93	.32**	.47**	.39**	.45**	.35**
Health and recreation behavior	0.98	0.82	.31**	.47**	.44**	.48**	.39**
13. Ability to relax	17.12	1.78	.16**	.30**	.33**	.32**	.26**
14. Active recreation	17.74	1.65	.31**	.44**	.34**	.36**	.31**
15. Health prevention	17.39	1.95	.23**	.28**	.31**	.41**	.31**

*Note. N* = 1116.

**p < .01.

### Shared Variance of the Personality Inventories

To evaluate the shared variance of the personality inventories, we performed several regression analyses. First, the Big Five were regressed on the IPS domains (Table 2a) and vice versa (Table 2b). Second, the Big Five were regressed on the IPS facets (Table 3). Third, the IPS facets were regressed on the Big Five (Table 4). The domain-wise regressions (Table 2a) indicated that 60% of *social and communicative behavior* could be predicted by the Big Five with extraversion being the strongest predictor (β = .51). 51% of *achievement behavior* could be predicted by the Big Five, especially by emotional stability (β = .32) and conscientiousness (β = .27). Regarding *health and recreation behavior*, the Big Five only explained 37% of the variance (conscientiousness: β = .26, extraversion: β = .24). The reverse regression analyses showed that the IPS domains could account for 15% (openness) to 50% (extraversion) of the variance in the Big Five. Extraversion was predicted best by *social and communicative behavior* (β = .61), whereas *achievement behavior* (β = .36) and *social and communicative behavior* (β = .27) were the best predictors of emotional stability. For conscientiousness and openness, *achievement behavior* was the best predictor (β = .28 and β = .20). Agreeableness could be predicted to similar extent by the three IPS domains (.15 ≤ β ≤ .19).

**Table 2a:** Summary of Regression Analyses of the Big Five on the IPS Domains

Construct	*R^2^*	*R^2^_adj_*	β				
			Openness	Conscientiousness	Extraversion	Agreeableness	Emotional Stability
Social and communicative behavior	.60	.60	.03	.19**	.51**	.08**	.19**
Achievement behavior	.51	.51	.10**	.27**	.24**	.04	.32**
Health and recreation behavior	.37	.37	.07**	.26**	.24**	.12**	.16**

*Note. N* = 1116.

**p < .01. *p < .05.

**Table 2b:** Summary of Regression Analyses of the IPS Domains on the Big Five

Construct	*R^2^*	*R^2^_adj_*	β		
			Social and communicative behavior	Achievement behavior	Health and recreation behavior
Openness	.15	.15	.13**	.20**	.11**
Conscientiousness	.33	.33	.16**	.28**	.22**
Extraversion	.50	.50	.61**	.08*	.06*
Agreeableness	.21	.21	.18**	.15**	.19**
Emotional Stability	.39	.39	.27**	.36**	.08*

*Note. N* = 1116.

**p < .01. *p < .05.

**Table 3:** Summary of Regression Analyses of the Big Five on the IPS Facets

IPS Facet	*R^2^*	*R^2^_adj_*	β				
			Openness	Conscientiousness	Extraversion	Agreeableness	Emotional Stability
Social and communicative behavior							
1. Activity	.31	.31	-.03	-.01	.60**	.04	-.08**
2. Assertiveness	.48	.47	.00	.09**	.61**	.01	.08**
3. Confrontation	.38	.38	-.03	-.21**	.02	-.37**	-.18**
4. Leadership	.20	.19	.07*	.20**	.26**	-.28**	.14**
5. Considerateness	.20	.19	.08**	.08*	.11**	.37**	-.10**
6. Sensitivity	.32	.31	-.03	-.11	-.13	-.04	-.39**
Achievement behavior							
7. Commitment	.26	.26	.03	.33**	.16**	.06	.09**
8. Inertia	.23	.23	-.04	-.11**	-.16**	-.05	-.27**
9. Stability	.25	.24	.13**	.19**	.07*	.02	.27**
10. Self-confidence	.26	.25	.03	.09**	.10**	-.04	.41**
11. Career commitment	.22	.22	.09**	.17**	.26**	.00	.11**
12. Optimism	.34	.34	.10**	.25**	.27**	.09**	.10**
Health and recreation behavior							
13. Ability to relax	.17	.17	-.01	.18**	.14**	.06	.17**
14. Active recreation	.27	.27	.11**	.15**	.28**	.10**	.07*
15. Health prevention	.22	.21	.07*	.28**	.06	.09**	.12**

*Note. N* = 1116.

**p < .01. *p < .05.

**Table 4:** Summary of Regression Analyses of the IPS Facets on the Big Five

Big Five Dimension	*R^2^*	*R^2^_adj_*	β														
			Social and communicative behavior						Achievement behavior						Health and recreation behavior		
			Activity	Assertiveness	Confrontation	Leadership	Considerateness	Sensitivity	Commitment	Inertia	Stability	Self-confidence	Career commitment	Optimism	Ability to relax	Active recreation	Health prevention
Openness	.19	.18	-.01	.06	-.01	.06	.09**	-.01	.00	-.03	.12**	-.03	.09**	.12**	-.06	.13**	.04
Conscientiousness	.41	.40	-.04	.01	-.19**	.07*	.06*	.00	.21**	-.01	.05	-.04	.04	.15**	.06*	.05	.15**
Extraversion	.58	.58	.25**	.39**	.03	.08**	.05*	-.08**	.00	-.07	.00	-.01	.04	.13**	.01	.07**	-.05*
Agreeableness	.42	.41	.06*	-.01	-.36**	-.17**	.22**	-.06*	.04	-.02	.02	-.06*	.01	.08**	.03	.08**	.07*
Emotional Stability	.44	.43	.00	.11**	-.12**	.05*	-.02	-.21**	.00	-.08**	.10**	.19**	-.01	.05	.06*	.02	.02

*Note. N* = 1116.

**p < .01. *p < .05.

In Table 3, the Big Five were regressed on the IPS facets. Although most IPS facets were significant predictors of the Big Five, most beta coefficients were rather low. The IPS facets accounted for 19% (openness) to 58% (extraversion) of variance in the Big Five. Results in Table 4 show that the Big Five accounted for 20 to 48% of the variance in the IPS facets within the domain *social and communicative behavior.* Between 22 and 34% of variance in the *achievement behavior* and 17 to 27% of variance in the *health and recreation behavior* facets could be explained by the Big Five.

### Criterion Validity

The satisfaction measure was substantially negatively skewed and leptokurtic (*S* = -1.28, *z = -*7.17, *p* < .01; *K =* 1.29, *z =* 3.62, *p* < .01) and academic achievement was platykurtic (*S* = -.19, *z = -*0.21, *p* > .05; *K = -*.45, *z = -*2.42, *p* < .05). We hence used non-parametric correlations (*Spearman*) throughout the validity analyses. Academic achievement and study satisfaction were positively correlated (*r_s_ = .*27, *p* < .05).

Regarding academic achievement, the Big 5 traits conscientiousness (*r_s_* = .26, *p* < .01), openness (*r_s_* = .11, *p* < .01) and agreeableness (*r_s_* = .10, *p* < .05) as well as the IPS facets *optimism* (*r_s_* = .09, *p* < .05) and *sensitivity* (*r_s_* = .08, *p* < .05) showed positive correlations. Predicting satisfaction, the broad IPS domain *achievement behavior* (*r_s_* = .18, *p* < .05) as well as the Big Five factor emotional stability (*r_s_* = .17, *p* < .05) were correlated; on the narrow level, only *career commitment* (*r_s_* = .22, *p* < .01) was correlated with academic achievement. To examine the predictive validity, we calculated a structural equation model (SEM) for each criterion^ii^. Conscientiousness (β = .26) and *sensitivity* (β = .17) could explain 9% of variance in academic achievement (χ^2^[11] = 31.21, *p* = .02; CFI = .98, RMSEA = .027, SRMR = .020). For satisfaction (*R*^2^ = 4%; χ^2^[6] = 11.45, *p* = .08; CFI = .96, RMSEA = .029, SRMR = .056), only *career commitment* (β = .22) was a signifiant predictor.

## Discussion

It could be shown that the broad Big Five traits and the narrow IPS traits share variance and are able to predict each other at varying degrees. Therefore, the question of whether both questionnaires should be used in a (teacher) student selection assessment can clearly be answered with a “No” for economic reasons. However, both questionnaires have their own advantages and provide unique contributions to the prediction of study satisfaction and academic achievement.

### Arguing the Case for the Big Five

#### The Big Five Predict Two of the Three IPS Domains Well

The regression analyses showed that the Big Five (mainly extraversion) accounted for 60% of the variance in *social and communicative behavior*. In particular, the IPS facets *activity* and *assertiveness* can be predicted to a large extent by the broad trait extraversion (.60 ≤ β ≤ 61). Consistent with our hypotheses, *achievement behavior* was associated with emotional stability and conscientiousness. About 50% of the variance in *achievement behavior* could be explained by the Big Five, particularly by emotional stability and conscientiousness. The amount of prediction was similarly high for all facets of *achievement behavior* (.22 ≤ *R*^2^ ≤ .34). Only *health and recreation behavior* was predicted worse by the Big Five (37%).

By contrast, the IPS scales do not predict the Big Five very well. Variance in openness and agreeableness could only be explained poorly by the IPS domains (.15 ≤ *R*^2^ ≤ .21). However, when using the IPS facets, agreeableness could be predicted much better (*R*^2^ = .42). Especially the facet *confrontation* accounted for a considerable amount of variance (β = -.37). Regarding openness, the amount of explained variance only increased marginally when considering the IPS facets (*R*^2^ = .19).

### Arguing the Case for the IPS

#### The IPS Is Able to Predict Important Aspects of the Big Five

Approximately half of the Big Five trait extraversion’s variance could be explained by the IPS domains (mainly by *social and communicative behavior*). On the IPS facet level, *assertiveness* was the best predictor of extraversion (β = .38). Approximately one third of variance in emotional stability and conscientiousness each could be explained by the IPS domains. For emotional stability, *social and communicative behavior* (with the facet *sensitivity*) as well as *achievement behavior* (with the facet *self-confidence*) were predictive. Conscientiousness could be predicted by all three IPS domains, but on the facet level, only *confrontation* and *commitment* reached considerably high betas.

However, one IPS domain could not sufficiently be explained by the Big Five. The domain *health and recreation behavior* was predicted worst by the Big Five; only 37% of its variance could be explained. Counterintuitively, it was marginally associated with emotional stability but instead higher with conscientiousness and extraversion. On the facet level, not every facet of the IPS could sufficiently be covered by the Big Five: for example, only 20% of variance in the facet *efficacy in leadership role* – undoubtedly a key characteristic in a teacher (Little & Akin-Little, 2008) – could be explained.

### Validity

Regarding academic achievement, only the broad Big Five traits conscientiousness and openness showed substantial correlations; the correlations of the narrow traits, however, were negligible. Explaining achievement using SEM, only conscientiousness remained a significant predictor. These results support the use of the Big Five. Additionally, the well-established Big Five provide an economically efficient assessment of personality traits that are relevant for multiple areas of behavior (Paunonen & Ashton, 2001). With respect to academic satisfaction, the broad traits emotional stability and *achievement behavior* as well as the narrow IPS trait *career commitment* were significantly correlated. SEM results indicated a predictive advantage of narrow traits: only *career commitment* could explain variance in satisfaction, which argues in favor of including narrow traits. The IPS offers another advantage: it does not only assess general behavior tendencies but also takes the situational context into account. A benefit of situation-specific tests is the *social validity* and acceptance by testees. For example, media may argue whether young people applying for teacher education should uncover their attitudes about seemingly job-irrelevant issues as they are usually assessed in broad Big Five questionnaires. In this context, including a construct such as *social and communicative behavior* as a proxy for the daily life of a teacher could be accepted better because of higher *face validity*. Questionnaires with higher face validity also lead to less faking and have been shown to be better predictors of later long-term achievements (Holden & Jackson, 1979; Lievens, Peeters, & Schollaert, 2008). Interpreting the criterion validity results, we need to consider that the amount of variance explained by personality predictors was generally low regarding the criterion satisfaction (only 4%).

### Limitations

Apart from these promising results, we need to mention some limitations of our study. Although the main sample was large, the follow-up sample for examining academic satisfaction was smaller than expected. The response rate for the questionnaire was approximately 27%. For future studies, we might prefer testing in regular college lessons with required presence to increase the follow-up sample. This would not only lead to a larger sample but also to a better representation of those who are not as satisfied with their studies. It would also involve people who typically do not engage in online surveys. This might enhance the generalizability of the results.

One part of our data was gathered in the actual admission exam. Even though field studies offer important advantages over laboratory studies, we need to pay attention to one disadvantage: the possible effect of socially desired response behavior in the admission procedure could lead to higher correlations and overlaps. Based on Krammer and Pflanzl (2015), who examined the effects of three different instructions (honest instruction, instruction to reproduce the faking of the admission exam and “*faking-good*”-instruction) on the answer behavior, our future studies could examine how social desirability influences the overlap of personality constructs.

Academic performance was already examined after the fourth semester. This allows a first estimation of the relationship between personality and academic achievement. However, the performance should be evaluated again in the future. Furthermore, other criteria of academic and job performance should be investigated, including practical grades, classroom management and pedagogical knowledge. For the evaluation of the admission exam, we also need to examine the relationship with actual job performance and real-life dropout. Follow-ups in the sixth semester as well as after one year in the teaching profession are planned.

Finally, potential mediators of the relationship between personality and academic success should be included. Models on student dropout (Tinto, 1975) and teacher job achievement (Helmke, 2006; Mayr, 2012) argue that the influence of personality on later achievement is mediated by factors such as the use of learning environments, motives and self-efficacy.

### Conclusions

The BFI-42 is a broad and comprehensive personality assessment, the IPS a narrower but more fine-grained assessment. The Big Five offer a broad picture of an individual’s personality as a whole. The IPS, by contrast, focuses on more specific aspects, namely on the manifestation of certain personality traits in different situations. The comparison of the different regression analyses – the IPS regressed on the Big Five and vice versa – showed inconsistent results. The IPS domains could be predicted better by the Big Five than the Big Five by the IPS domains. On the facet level, we found contrary results: the IPS facets could be explained worse by the Big Five than vice versa. However, most of the facets are only predictive of one or two Big Five traits and to a relatively low extent. This supports the assumption that aggregation is accompanied by a loss of specific variance (Paunonen, 1998). In addition, the prediction of the global Big Five traits by 15 narrow IPS facets does not seem very economic.

Apart from globally comparing the two personality tests, specific domains and facets can be related. S*ocial and communicative behavior* shared a large amount of variance with extraversion as well as *achievement behavior* with conscientiousness and emotional stability. When comparing the variance overlaps of these constructs, we found that the Big Five factors could be predicted to a larger extent by the IPS facets than vice versa. That means that we face a greater loss of information if the narrow traits are omitted in favor of the broad traits. However, the opposite is true when comparing the Big Five factors with the IPS domains: here, the Big Five can account for more variability in the IPS domains than vice versa.

However, there is one important exception to this rule: the domain *health and recreation behavior* could not be adequately covered by the Big Five. However, especially this domain seems to be of high importance in professions that are particularly prone to burnout and related health problems, as it is the case with teachers (Albisser, Kirchhoff, & Albisser, 2009). In addition, there is evidence for a relationship between *health and recreation behavior* and teaching achievement already in college (Krammer et al., 2016). Our study offers some evidence which of these narrow traits (e.g. *career commitment*) might be relevant for predicting academic achievement and satisfaction in teacher studies. Further studies using our data to also assess later teacher success and satisfaction are planned.

Our findings can be seen as support for the concept of the bandwidth-fidelity dilemma: broad predictors should be used for a complex and diverse occupational profile but also complemented with narrower traits (here: a construct comparable to the domain *health and recreation behavior)* when testing for very specific criteria, for example, job-satisfaction or burnout-risk, in a specific field, such as teacher student admission tests.
